# Retraction: Expression of DNMT1 and DNMT3a Are Regulated by GLI1 in Human Pancreatic Cancer

**DOI:** 10.1371/journal.pone.0294385

**Published:** 2023-11-09

**Authors:** The PLOS ONE Editors

Following the publication of this article [1], concerns were raised regarding results presented in Figs. 1, 4, 6, and 7. Specifically,

- The Fig. 1 DNMT1 Normal tissue panel and DNMT3a Normal tissue panels appear to partially overlap.
- Vertical irregularities suggestive of splice lines were detected in the following figures:
  - Fig. 4D DNMT3a panel
  - Fig. 7 ACP and hMLH1 panels
- In Fig. 6i, when levels are adjusted to visualize background, there appears to be a splice line between the Marker and the Input lanes, and it appears as though a uniform gray/black box overlays a large area of lanes 5 and 6.

During editorial follow-up, the first author stated that they were unable to provide the original data underlying the published results and requested retraction of the article. PLOS did not receive a response from the corresponding author. In the absence of the underlying data, the concerns with this article cannot be resolved.

In light of the concerns affecting results presented in Figs. 1 and 6 that question the reliability and integrity of these data, the *PLOS ONE* Editors retract this article.

SSH agreed with the retraction but stands by the article’s findings. FW, LJW, CYG, RW, AWK, LX, GYH, XFX, JS, and XPW either did not respond directly or could not be reached.
